# Identification of Novel Variants of Thyroid Hormone Receptor Interaction Protein 13 That Cause Female Infertility Characterized by Zygotic Cleavage Failure

**DOI:** 10.3389/fphys.2022.899149

**Published:** 2022-06-23

**Authors:** Huiling Hu, Shuoping Zhang, Jing Guo, Fei Meng, Xueqin Chen, Fei Gong, Guangxiu Lu, Wei Zheng, Ge Lin

**Affiliations:** ^1^ Laboratory of Reproductive and Stem Cell Engineering, NHC Key Laboratory of Human Stem Cell and Reproductive Engineering, Central South University, Changsha, China; ^2^ Clinical Research Center for Reproduction and Genetics in Hunan Province, Reproductive and Genetic Hospital of CITIC-Xiangya, Changsha, China

**Keywords:** female infertility, variants, *TRIP13*, zygotic cleavage failure, DNA damage

## Abstract

Zygotic cleavage failure (ZCF) is a severe, early type of embryonic arrest in which zygotes cannot complete the first cleavage. Although mutations in *BTG4* and *CHEK1* have been identified as genetic causes of ZCF, these genes only explain a small population of ZCF cases. Thus, the underlying genetic causes for other affected individuals need to be identified. Here, we identified three *TRIP13* missense variants responsible for ZCF in two patients and showed that they followed a recessive inheritance pattern. All three variants resulted in obvious changes in hydrogen bonding and consistent increase in DNA damage. Additionally, transcriptomic sequencing of oocytes and arrested embryos containing these variants suggested a greater number of differentially expressed transcripts in germinal vesicle (GV) oocytes than in 1-cell embryos. Vital genes for energy metabolism and cell cycle procession were widely and markedly downregulated, while DNA repair-related genes were significantly upregulated in both GV oocytes and 1-cell embryos of patients. These findings highlight a critical role of *TRIP13* in meiosis and mitosis, as well as expand the genetic and phenotypic spectra of *TR1P13* variants with respect to female infertility, especially in relation to ZCF.

## Introduction

Infertility affects approximately 48 million females worldwide and is routinely treated using *in vitro* fertilization (IVF) or intracytoplasmic sperm injection (ICSI) (Mascarenhas et al., 2012; Chambers et al., 2021). However, some females experience recurrent IVF/ICSI failure because of oocyte meiotic arrest (OMA), total fertilization failure, and early embryonic arrest (EEA), and EEA is the most common abnormality (Betts and Madan, 2008; Sang et al., 2021).

In the first cytokinesis of the zygote, the pronuclear envelopes break down and maternal and paternal chromosomes align at the metaphase plate mediated by the bipolar mitotic spindle (Li et al., 2013). Metaphase chromosomes are arranged on the equatorial plate, forming mitotic checkpoint complexes (MCCs); only when each spindle microtubule is properly connected to the centromere can the MCC be separated and anaphase be initiated (Lara-Gonzalez et al., 2012; Faesen et al., 2017). Subsequently, the zygotes are converted from the 1-cell to the 2-cell stage and then undergo consecutive occurrences of cytokinesis, producing six to eight blastomeres on day 3 of cultivation (Niakan et al., 2012).

Zygotic cleavage failure (ZCF) is a unique early type of EEA in which successfully fertilized zygotes cannot complete the first cleavage and remain arrested in the 1-cell stage. We defined this phenotype as a Mendelian disease for the first time in 2020, and identified variants in the B-cell translocation gene-4 (*BTG4*) as a genetic cause for maternal mRNA decay and ZCF (Zheng et al., 2020). In 2021, dominant variants in checkpoint kinase 1 (*CHEK1*) were proven to cause G2/M transition failure and ZCF owing to overactivation of kinase activity (Chen et al., 2021; Zhang et al., 2021). However, known variants in *BTG4* and *CHEK1* only account for 20% of the ZCF population (Zhang et al., 2021), and the underlying genetic causes for other affected individuals remain largely unknown.

Thyroid hormone receptor interaction protein 13 (*TRIP13*) functions as an important regulatory molecule of MCC dissociation and chromosome recombination in meiosis (Li and Schimenti, 2007; Wang et al., 2014). Female mice lacking *TRIP13* display a severely impaired ovarian reserve (Li and Schimenti, 2007), and biallelic pathogenic variants of *TRIP13* reportedly cause OMA and female infertility (Zhang et al., 2020). However, it is unknown whether there is any other phenotype related to female infertility, thus meriting further investigation.

Here, we report two newly discovered *TRIP13* variants (c.1141G > A, p. Glu381Lys and c.1258A > G, p. Lys420Glu), and one recurrent previously reported *TRIP13* variant (c.77A > G, p. His26Arg), resulting in phenotypic variability. This study suggests a novel, possible genetic pathogenesis of ZCF and extends the spectrum of phenotypes for *TRIP13* variants.

## Materials and Methods

### Ethical Approval and Patient Consent

Infertile patients diagnosed with ZCF were recruited from the Reproductive and Genetic Hospital of CITIC-XIANGYA. All blood samples, immature germinal vesicle (GV) oocytes, and arrested embryos were donated for the study after written informed consent from the participants.

### Whole Exome Sequencing and Variant Analysis

Genomic DNA was extracted from peripheral blood samples using a QIAamp DNA Blood Kit (Qiagen, 51106, Germany) following the manufacturer’s protocol. Exome capture and sequencing were performed using xGen Exome Research Panel v1.0 and the Illumina HiSeq 2500 platform. Single nucleotide variants (SNVs) and small insertions and deletions (indels) were generated with the Genome Analysis Toolkit (GATK) pipeline and annotated with ANNOVAR. Variants were filtered, as previously described (Zheng et al., 2020), using the following criteria: 1) variants with minor allele frequencies (<1% in the Genome Aggregation Database); 2) exonic nonsynonymous or splice site variants, or coding insertions or deletions (indels); 3) mRNA and/or proteins that were highly or specifically expressed in oocytes; 4) coexistence in at least two probands, but absence in fertile populations.

### *In Silico* Analysis

Wild-type (WT) and *TRIP13* variants were assessed using the SWISS-MODEL software (https://swissmodel.expasy.org) based on the 6f0x.1.C.pdb template. Variants were mapped to the atomic model using PyMol (http://www.pymol.org). Evolutionary conservation analysis was performed with DNAMAN software.

### Cell Culture and Transfection

HeLa cells were cultured in Dulbecco’s Modified Eagle Medium (DMEM)/high glucose (Hyclone, SH3002201) with 10% fetal bovine serum (Hyclone, SH30070) in a humidified incubator at 37°C with 5% CO_2_. Transient transfections were performed using Lipofectamine 3000 (Thermo Fisher Scientific, L3000015) according to the manufacturer’s instructions. Approximately 48 h after transfection, cells were fixed for immunofluorescence.

### Immunofluorescence and Immunoblotting

HeLa cells were fixed in 4% paraformaldehyde for 20 min at room temperature and permeated with 0.1% Triton X-100 for 15 min. After blocking for 1 h with 4% bovine serum albumin (A600332-0100, Sangon Biotech), samples were incubated with primary anti-FLAG (1:500 dilution, 701629, Invitrogen), anti-γH2Ax (1:500 dilution, 9718S, Cell signaling technology) and corresponding donkey anti-mouse Ig (H + G) (1:1000 dilution, A21203, Alexa Flour) antibodies. Hoechst 33342 (Invitrogen, H3570, 1:1000 dilution) was used to stain the nucleus. Four groups of cells were imaged using the same magnification on the same day to compensate for experimental error.

Cells were lysed with RIPA lysis buffer (Strong) (P0013E-1, Beyotime) and proteins denatured with 4× sodium dodecyl sulphate (SDS) loading buffer (NP0007, Invitrogen). The proteins were separated by SDS-polyacrylamide gel electrophoresis (SDS-PAGE Gel Kit, CW0022M, CWBIO), then transferred to a PVDF membrane. After blocking with 5% non-fat milk in Tris-HCl buffer solution with Tween-20 (TBST), the PVDF membrane was incubated with primary antibodies overnight at 4 °C (anti-FLAG, 1:1000 dilution, 8146S, Cell Signaling; and anti-GAPDH, 1:1000 dilution, AF0006, Beyotime). After washing three times with TBST, membranes were incubated with goat anti-mouse IgG (1:4000 dilution, A0216, Beyotime) to detect the primary antibodies.

### Single Oocyte/Embryo RNA Sequencing and Data Analysis

For RNA sequencing (RNA-seq), the following samples were collected: 1) seven GV oocytes from patient 1 and ten GV oocytes from a control patient, from whom several embryos were collected for transfer during assisted reproductive technology, and 2) three 1-cell stage arrested embryos each from patient 1 and patient 2, and seven control 1-cell embryos of which previously described (Leng et al., 2019). RNA-seq libraries were prepared using the SMARTSeq2 protocol, sequenced using the BGISEQ500 platform (BGI-Shenzhen), and the data analyzed as previously described (Leng et al., 2019; Sha et al., 2020). Gene expression levels were normalized as transcripts per million (TPM). Transcripts whose levels increased or decreased by more than 2-fold were considered as differentially expressed.

## Result

### Clinical Characteristics of Patients

**FIGURE 1:**
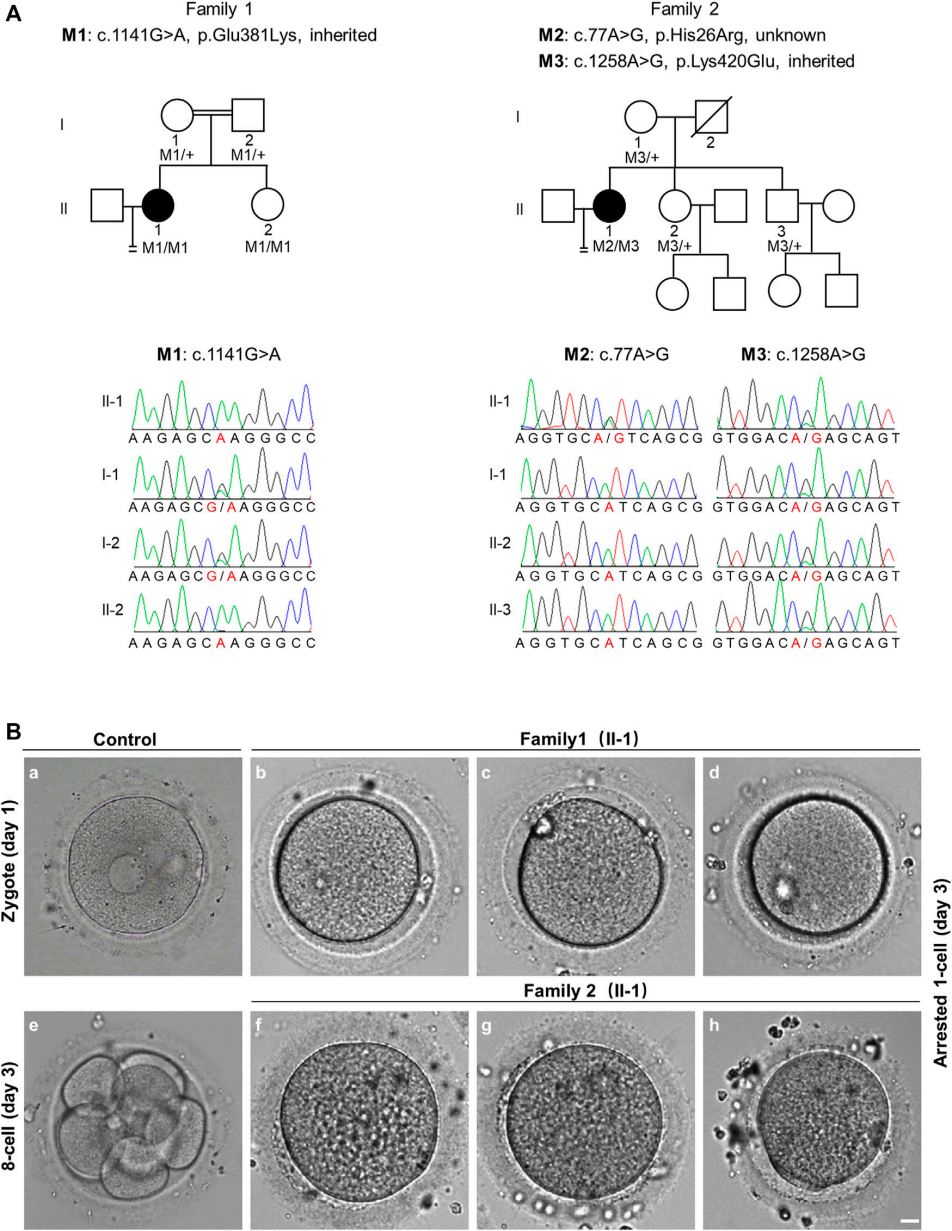
Pedigree-based identification of *TRIP13* variants and the zygotic cleavage failure (ZCF) phenotype. **(A)** Genetic analysis of two patients from different families affected by ZCF. Black circles indicate the affected individuals. Sanger sequencing confirmation is shown below the pedigrees. **(B)** Morphology of a control embryo and arrested embryos from both patients on day 3. All six arrested 1-cell embryos were from two-pronucleus fertilized oocytes. Scale bar = 10 μm.

Patient 1 from a consanguineous family, was 29 years old and diagnosed with primary infertility for unexplained reasons at 25 years of age. Her parents were first cousins. During her previous two ICSI attempts, 43 oocytes were retrieved, of which 25 were arrested at the GV or MI stage, and the other 18 mature MII oocytes formed 7 zygotes. Six zygotes remained at the 1-cell stage. Patient 2 was 36 years old, and experienced primary infertility for six years, despite a normal menstrual cycle and no endocrine disorders. In her previous three ICSI attempts, 6 of 13 oocytes retrieved were mature, of which 3 fertilized oocytes formed; however, they were arrested at the 1-cell stage (Table 1 and Figure 1B).

**TABLE 1:** Oocyte and embryo characteristics of ICSI attempts for the affected individuals.

Family NO.	Age (years)	DIF (years)	COH protocol	IVF/ICSI attempt	Retrieved Oocytes	Immature Oocytes	MII Oocytes	Fertilized Oocytes	Cleaved embryos
1	29	4	Long (initiated with 112.5 IU human rFSH; trigger with 5000 IU HCG)	ICSI	16	12	4	3	0
			GnRH-ant (initiated with 112.5 IU human rFSH; dual trigger with 5000 IU HCG and 0.2 mg GnRHa)	ICSI	27	13	14	4	1, yet arrested in 3-cell stage
2	36	6	Long (initiated with 250 IU human rFSH; trigger with 8500 IU HCG)	ICSI	8	5	3	2	0
			GnRH-ant (initiated with 300 IU human rFSH; dual trigger with 5000 IU HCG and 0.2 mg GnRHa)	ICSI	1	1	0	—	—
			Long (initiated with 300 IU human rFSH; trigger with 8500 IU HCG)	ICSI + AOA	4	1	3	1	0

Abbreviation: DIF, duration of infertility; COH, controlled ovarian hyperstimulation; GnRH-ant, gonadotropin-releasing hormone antagonist; HCG, human chorionic gonadotropin; rFSH, recombinant follicle stimulating hormone; ICSI, intracytoplasmic sperm injection; AOA, assisted oocyte activation.

### Identification of Variants in Thyroid Hormone Receptor Interaction Protein 13

Since patient 1 is from a consanguineous family, the homozygous variant was considered a priority. After data filtering, only one rare homozygous variant c.1141G > A, p. Glu381Lys in *TRIP13* was identified. This variant was inherited from her parents, and her sister carries the same homozygous variant; however, her fertility is not known owing to no sexual history. Patient 2 had the following compound heterozygous variants: c.77A > G, p. His26Arg and c.1258A > G, p. Lys420Glu. The c.1258A > G variant was inherited from her mother, and her fertile sister and brother also harbor this variant. However, the source of the c.77A > G variant is unknown owing to the unavailability of DNA sample from her father (Figure 1A and Table 2).

**TABLE 2:** Overview of the *TRIP13* variants observed in two families.

Probands in families	Genomic position on chr 5 (bp)	Exon	cDNA change	Protein change	Mutation type	Genotype	SIFT	Mutation taster	gnomAD AF
1	916026	12	c.1141G > A	p.Glu381Lys	Missense	Homozygous	Tolerated	Deleterious	8.12E-06
2	893190	1	c.77A > G	p.His26Arg	Missense	Compound-heterozygous	Tolerated	Neutral	9.37E-06
	917177	13	c.1258A > G	p.Lys420Glu	Missense		Tolerated	Deleterious	0

### Localization and Effect for Thyroid Hormone Receptor Interaction Protein 13 Variants in HeLa Cells

**FIGURE 2:**
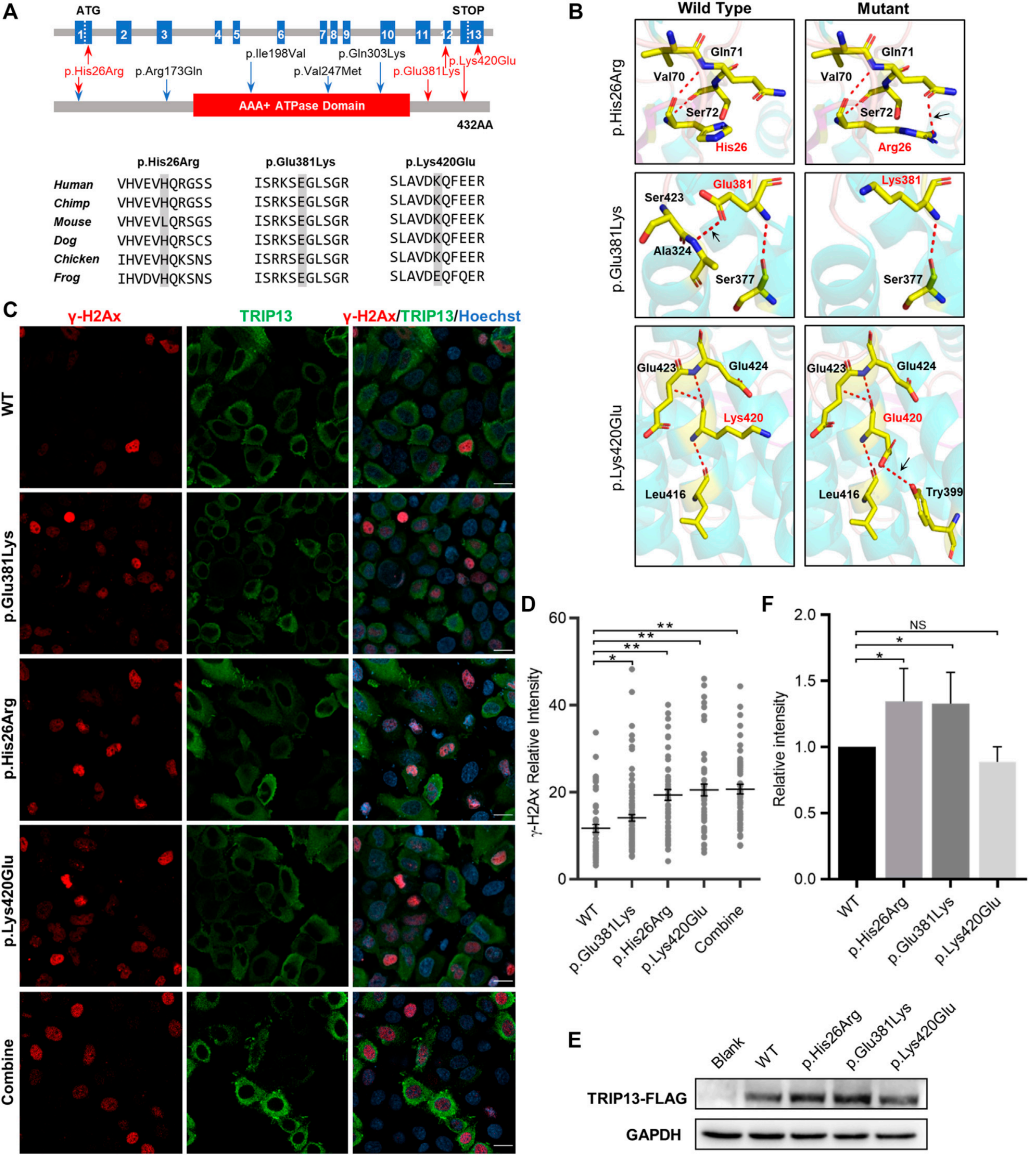
Effects of *TRIP13* variants on predicted conformation, location of TRIP13 protein and DNA damage level in HeLa cells. **(A)** Localization of variants in the genome (top) and in a schematic of the TRIP13 protein structure (middle), and conservation of mutated amino acids in the variants in six species (bottom). The red arrows indicate novel variants identified herein; blue arrows indicate previously reported variants. **(B)** Protein conformation predictions of *TRIP13* variants. Red dashed lines represent hydrogen bonds and black arrows indicate hydrogen bond changes. **(C)** Effects of *TRIP13* variants on cellular localization of TRIP13 protein and DNA damage level in transfected HeLa cells. “combine” indicated co-transfected the p. Glu381Lys and p.Lys420Glu plasmids into the HeLa cells. Scale bar = 20 μm. **(D)** Statistical analysis of γH2Ax intensity shown in panel **(C)**. Data are the mean ± SD. **(E)** Effects of *TRIP13* variants on TRIP13 protein levels in transfected HeLa cells. **(F)** Statistical analysis of TRIP13 protein abundance shown in panel **(E)**. The relative protein intensities of FLAG/GAPDH in the WT group were set as 1. Data are the mean ± SD.

All three TRIP13 variant residues herein are located in both sides of the ATPase domain; variant p. His26Arg is in the N-terminus and the other two variants are in the C-terminus. The variants p. Glu381Lys and p. Lys420Glu are highly conserved at the amino acid level among six different species, whereas the variant p. His26Arg is not conserved in mouse (Figure 2A). None of the three variants had any obvious effect on the TRIP13 protein structure, but they did result in a loss of hydrogen bonding (as p.Glu381Lys) or the formation of new hydrogen bonds (as p.His26Arg and p.Lys420Glu) with nearby amino acids, based on the constructed three-dimensional structure (Figure 2B).

We examined the subcellular localization of TRIP13 and DNA damage level by immunofluorescence and its abundance by western blotting in transfected HeLa cells. Both the wild-type and mutant TRIP13 proteins were localized in the cytoplasm, with no obvious difference (Figure 2C). However, all mutant TRIP13 caused the γH2Ax (a classical DNA damage marker) signals accumulation (Figures 2C,D). However, compared with wild-type TRIP13 protein abundance, the variants p. Glu381Lys and p. His26Arg showed a significant increase in TRIP13 levels, whereas variant p.Lys420Glu showed a decrease trend (Figures 2E,F).

### Changes in Oocyte and Embryo Transcriptomes in the Thyroid Hormone Receptor Interaction Protein 13 Variants

**FIGURE 3:**
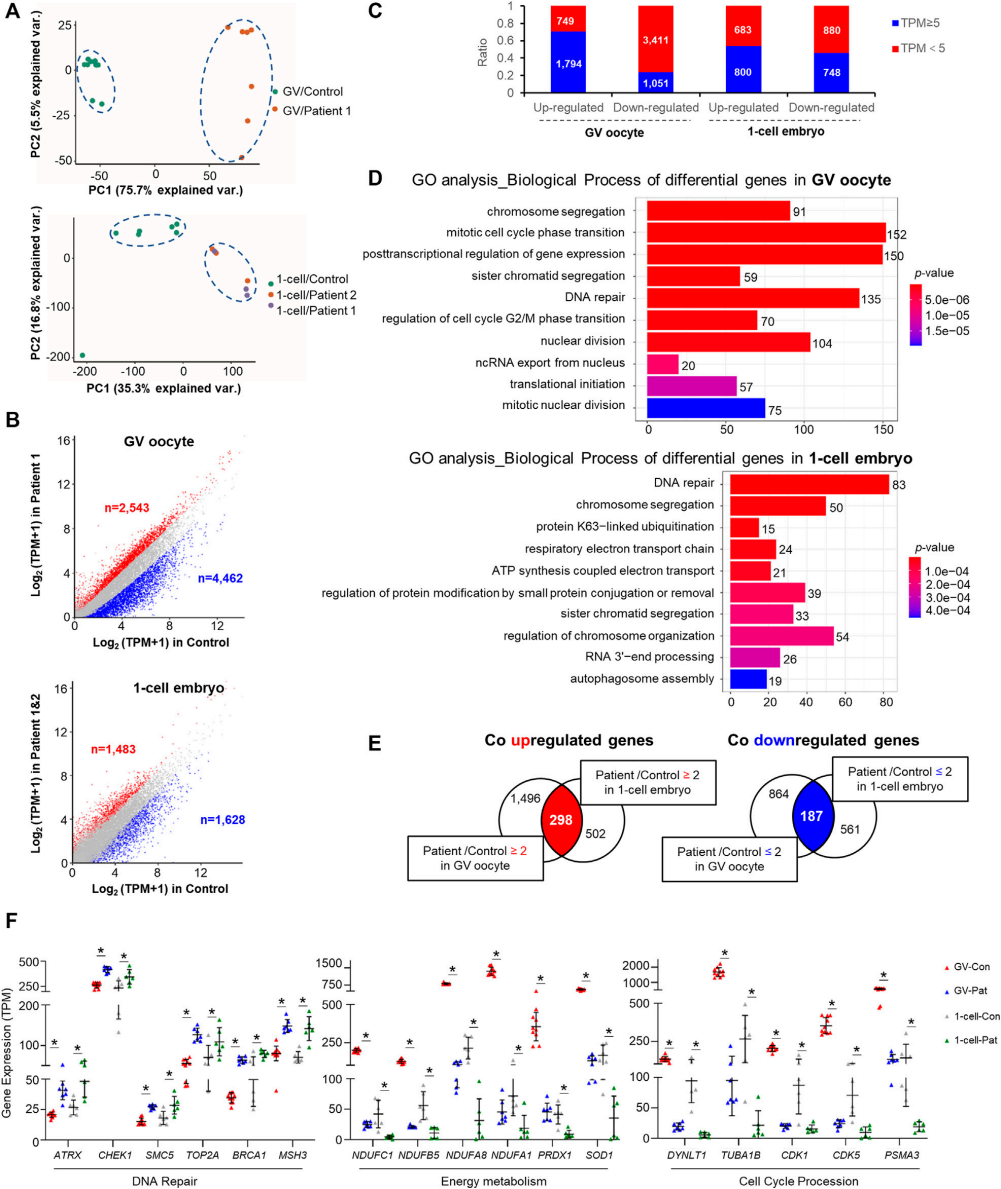
Changes in oocyte and embryo transcriptomes in the *TRIP13* variants. **(A)** Principal component analysis (PCA) of gene expression in germinal vesicle (GV) oocytes and 1-cell embryos of *TRIP13* variants and controls. **(B)** Scatter plot comparing the number of upregulated and downregulated gene transcripts between the *TRIP13* variant in patient and control GV oocytes (upper) or 1-cell embryos (lower). Transcripts whose levels increased or decreased by more than 2-fold in the *TRIP13* variant patients are highlighted in red or blue, respectively. **(C)** Ratio of the number of differentially expressed transcripts based on transcripts per million (TPM) prevalence in GV oocytes and 1-cell embryos. **(D)** Enriched top 10 biological processes of differentially expressed transcripts in GV oocytes and 1-cell embryos obtained via Gene Ontology (GO) analysis. Transcript numbers for each biological process are provided on the right. **(E)** Venn diagrams showing the shared upregulated (red region) or downregulated (blue region) transcripts between the control and GV oocytes (left) or 1-cell embryos (right). **(F)** Scatter plots of mRNA expression of selected transcripts related to three functions in the *TRIP13* variants and control groups. Data are the mean ± SD. *p < 0.01, Student’s t-test.

Principal component analysis revealed an appreciable distinction among the controls and patients (Figure 3A). There were 7,005 and 3,111 differentially expressed transcripts (>2-fold change) in GV oocytes and 1-cell stage embryos of patients with *TRIP13* variants, respectively (Supplementary Tables S1, S2). More transcripts were downregulated than upregulated in the GV oocytes of patient than in the controls (n_downregulated_ vs. n_upregulated_ = 4,462 vs. 2,543; 1.7-fold difference); however, a similar number of transcripts were downregulated and upregulated in 1-cell stage embryos of patients compared with the controls (n_downregulated_ vs. n_upregulated_ *=* 1,628 vs. 1,483) (Figure 3B). Additionally, more transcripts with a high abundance (TPM ≥ 5) were observed in the upregulated transcripts than in the downregulated transcripts, especially in the GV oocytes (Figure 3C).

### Abnormal Cell Cycle Progression in Oocytes and Zygotes of Affected Patients

Gene Ontology analysis revealed that the differentially expressed transcripts in GV oocytes and 1-cell embryos were consistently enriched in chromosome segregation and DNA repair process as the top 10 enriched biological processes (BPs) (Figure 3D). The other BPs were cell cycle phase transition, translation initiation, and nucleus division in GV oocyte and protein ubiquitination/modification, energy metabolism, and mRNA decay in 1-cell embryo. Based on the differences in biological events between GV oocytes and the 1-cell stage embryos, we investigated the commonalities between the two groups of regulated genes, identifying 298 co-upregulated and 187 co-downregulated transcripts among the high abundance differentially expressed genes (Figure 3E). Among the co-differentially expressed transcripts, we found that several genes responsible for DNA repair were highly expressed, whereas those involved in energy metabolism or cell cycle procession were significantly decreased in GV oocytes and 1-cell embryos of patients (Figure 3F). These results indicate that *TRIP13* may repair DNA damage and maintain energy support to promote cell cycle progression during human oocyte maturation and mitosis initiation.

## Disscusion

TRIP13 is a cell cycle regulator involved in both meiosis and mitosis (Li and Schimenti, 2007; Roig et al., 2010; Wang et al., 2014; Ma and Poon, 2016; Zhang et al., 2020). In this study, we report three different missense variants in *TRIP13* that are responsible for human ZCF, which followed a recessive inheritance pattern.

Zhang et al. (2020) identified five variants in *TRIP13* responsible for OMA and reported that two independent individuals who experienced OMA carried c.77A > G homozygous variant (Zhang et al., 2020). However, here, variants c.77A > G and c.1258A > G constituted a compound heterozygous pattern, and this patient exhibited a milder abnormality, with half the retrieved oocytes being mature. This phenotypic heterogeneity may suggest that the increase in TRIP13 protein abundance caused by c.77A > G was partially neutralized by the decrease in TRIP13 protein abundance caused by c.1258A > G (Figure 2F). However, because of a scarcity of human oocytes and embryos, the exact protein abundance in oocytes or embryos is largely unknown.

Yost et al. (2017) reported that the homozygous nonsense variant c.1060C > T (p.Arg354*) and the splicing c.673-1G > C pathogenic variant are both related to cause Wilms tumors (Yost et al., 2017). The differentially expressed transcripts in GV oocytes and 1-cell embryos were consistently enriched in chromosome segregation (Figure 3D), interestingly, chromosome segregation error is also the pathogenesis for Wilms tumors. However, both patients in our study suffered from infertility with no other diseases. The two distinct diseases, Wilms tumors and ZCF, might be caused by the state of the mutant protein. The Wilms tumor-associated variants caused the complete loss of the full-length protein, whereas the missense variants in the present study only affected the protein abundance.

Double strand breaks (DSBs) arise from endogenous and exogenous DNA damaging stimulation, persistent DSB could activate cell cycle checkpoints and resulted in cell cycle arrest (Mehta and Haber, 2014). Cells generally repair the DSB via homology-directed repair (HDR) or non-homologous end joining (NHEJ) to maintain the integrity of genomes (Chapman et al., 2012; Scully et al., 2019). TRIP13 directly activates DNA-dependent protein kinase catalytic subunit (DNA-PKcs) to activate the NHEJ pathway (Banerjee et al., 2014). We found the mutant TRIP13 plasmid transfection cause a more γH2Ax signals accumulation in HeLa cell (Figure 2D), additionally, the DNA repair pathway related genes significantly upregulated and cell cycle procession related genes significantly downregulated in both GV oocytes and 1-cell embryos of *TRIP13* mutant patients (Figure 3F), we suggested the mutant TRIP13 may reduce ability to repair DNA damage. However, because of a scarcity of human oocytes and embryos, the exact γH2Ax signals in oocytes or embryos cannot been visualized.

Global transcriptional silencing is a highly conserved event from fully grown oocytes to zygotic genome activation (ZGA) at 4–8 cell embryo stage (Yan et al., 2013; Sha et al., 2020). Thus, the GV oocyte is the highest point of maternal mRNA accumulation, followed mainly by mRNA decay until ZGA. We found a large number of differentially expressed transcripts in the GV oocyte phase, especially a high proportion of downregulated transcripts (Figure 3B), indicating that the effect of *TRIP13* variants was initiated during oocyte growth. Indeed, *Trip13* knockout mice exhibit total oocyte ovarian reserve loss, whereas *Trip13* hypomorphic variant mice still exhibit some growing follicles (Li and Schimenti, 2007; Pacheco et al., 2015). However, the ovarian reserve was not obviously affected in the patients in this study, which may be explained by the degree of *TRIP13* function loss.

Among the co-differentially expressed transcripts, *CHEK1* transcript levels were significantly increased in both oocytes and the 1-cell stage embryos of patients. *CHEK1* encodes a serine/threonine-protein kinase, which is required for cell cycle control. Dominant variants in *CHEK1* result in G2/M transition arrest of the zygote (Chen et al., 2021; Zhang et al., 2021). Here, we established a relationship between the two cell cycle regulators *TRIP13* and *CHEK1*. However, whether zygote arrest can be rescued by CHEK1 inhibitor treatment needs further investigation.

In summary, our study expanded the mutation and phenotype spectrum of *TRIP13* and suggested that human *TRIP13* biallelic variants can cause cell cycle progress abnormalities that result in female infertility characterized by human ZCF. These findings should facilitate genetic diagnosis of ZCF and guide the development of precise therapy in the future for clinical female infertility.

## Data Availability

The datasets presented in this article are not readily available because of local data protection laws. However, raw data is available from the corresponding authors on reasonable request.
